# Survey dataset on the types, prevalence and causes of deviant behavior among secondary school adolescents in some selected schools in Benin City, Edo State, Nigeria

**DOI:** 10.1016/j.dib.2018.07.059

**Published:** 2018-07-27

**Authors:** Sheila A. Bishop, Hilary I. Okagbue, Olumuyiwa A. Oludayo, Olasunmbo O. Agboola, Michael C. Agarana, Muminu O. Adamu

**Affiliations:** a Department of Mathematics, Covenant University, Ota, Nigeria; b Department of Business Management, Covenant University, Ota, Nigeria; c Department of Mathematics, University of Lagos, Akoka, Lagos, Nigeria

**Keywords:** Deviant behavior, Survey, Questionnaire, Statistics, Benin city, Survey analytics

## Abstract

This data article contains the exploratory analysis of data obtained from a field survey done to determine the types, prevalence and likely causes of deviant behaviors among secondary schools’ adolescents in some selected schools in Benin City. The data presents the findings in tables and will be helpful in childcare guidance, counseling, education management and for education policy makers.

**Specifications Table**

**Table:** 

Subject area	Social Sciences
More specific subject area	Guidance and Counseling, Child Psychology
Type of data	Tables
How data was acquired	Field Survey
Data format	Analyzed
Experimental factors	Simple random sampling of some selected secondary schools in Benin City, Nigeria.
Experimental features	Analysis of sample selection of the responses of students and teachers from structured Questionnaires.
Data source location	University of Benin, Edo State, Nigeria
Data accessibility	All the data are in this data article

**Value of the data**

• The data could be useful in detecting deviant behavior attributes in adolescents [1], [2].
• The data can be useful in child psychology.
• The data could be used by policy makers in formulating policies on child and adolescent mental health.
• The data can also help educationist in educational management especially in the area of curriculum and needed educational infrastructure.
• The data can also help secondary school teachers and lower level tertiary school teachers to predict and understand the behavior of their students and how to relate with them.
• The data can also help to shed more light on the possible solutions to the prevalence of such behaviors in similar demographics.
• Types of deviant behaviors exhibited by adolescents in different geopolitical zones can be compared. This can be very useful in making some decisions that have to do with the education of the child and well-being.

## 1 Data

The data is a set of responses solicited from a total of nine (9) randomly selected secondary schools consisting of private and public secondary schools (three mixed schools (MS), three boys’ schools (BS) and three girls’ schools (GS)) in Benin City, Nigeria. The mixed school can also be regarded as co-educational school while BS and GS can be regarded as same sex schools.

The data was obtained with the aid of structured questionnaires administered to the subjects (students and teachers). The investigators made two visits to each of these schools to ensure thorough examination and completion of each item. In each of these schools, hundred (100) students were randomly selected from both senior classes (SSS I, II, III) and junior classes (JSS I, II, III) making a total of nine hundred (900) students. On the other hand, fifteen (15) teachers were randomly selected from each of these schools making it a total of one hundred and thirty-five (135) teachers.

Thus, the sample of the data consists of nine hundred students and one hundred and thirty-five teachers.

The variables of the data are the measure of the deviant behavior of the respondents as responded by the students and teachers.

## 2 Experimental design, materials and methods

Different methodologies are available in the study of deviant (externalizing) and internalizing behavioral patterns in children and adolescents [3], [4], [5], [6], [7], [8], [9], [10]. Most often standardized or structured questionnaires tailored to suit particular (the studied) demographics are used [11], [12], [13], [14], [15]. Evolving trends and behavioral patterns are often observed and made available as scientific findings. Some examples can be seen in [16], [17], [18], [19], [20], [21], [22], [23], [24], [25], [26]. Different statistical analysis can be useful for further behavioral analysis [27], [28], [29], [30], [31], [32].

### 2.1 Instrument of data collection

The two instruments developed for this study are:

(i) Deviance Survey Scale for Students (DSSS)
(ii) Deviance Survey Scale for Teachers (DSST)

The DSSS consists of two parts. The first part is designed to obtain personal data from the students. The second part consists of two sections ‘A’ (consists of two items. Item 1 and 2 are designed to determine the types of deviant behaviors in such schools and the degree of their occurrence. The responses required are “Yes/No” and Rarely/Occasionally/ Very often.

Section ‘B’ consists of nine items, designed to determine the role or causes of these behaviors in secondary schools. The responses required are “Yes/No”. The DSSS can be assessed as Supplementary Data A**.**

The teacher’s questionnaire also consists of two parts. The first part is designed to obtain personal data from the selected teachers and the second part consists of three sections. Section ‘A’ is designed to determine the types of deviant behavior exhibited in such secondary schools and also the degree of their exhibition. The responses demanded are Rarely/ Occasionally/ Very often. Section ‘B’ consists of ten items, designed to determine the part played by parents, teachers, society, exposure to obscene films, social media, Face Book, twitter, Instagram, WhatsApp, literatures and school administrators as possible causes for the prevalence of deviant behaviors in secondary schools. The responses required are “Yes/No” responses.

In Section ‘B’ Item eleven (11) is designed to determine the measures used to check these behaviors from further reoccurrence, four (4) measures were listed, requesting the respondents to tick the ones used in their schools.

Item twelve (12) is a free response designed to solicit the teachers’ recommendation for solving the problems of deviant behavior so as to stop the prevalence and spread in our schools and society at large. The DSST can be assessed as Supplementary Data B**.**

The two questionnaires were validated with the help of experienced lecturers, school administrators, and senior tutors with respect to:

i. Comprehensiveness of each of the instruments in terms of the types of deviant behaviors and nature of their seriousness.
ii. The language in terms of clarity and meaningfulness to the students and teachers.

### 2.2 Method of data collection

Two-stage probability sampling was used to obtain the sampling frame and simple random sampling was used to administer the questionnaires. The choice of Benin City is because as the state capital, different demographics that constitute the state are captured. Non-response was reduced drastically because the investigators made two visits to each of these schools to ensure thorough examination and completion of each item.

The raw dataset for DSSS can be assessed as Supplementary Data C. The 21 variables used to measure the deviant behaviors are coded R1 to R21. Similarly, the one for DSST can be assessed as Supplementary Data D, where the variables as observed by the teachers (respondents) are coded S1 TO S21.

### 2.3 Data presentation

The total and mean score for the students and teachers which are the measure of the deviant behavior are presented in Table 1, Table 2 respectively.

**Table 1:** The data summary of analysis of DSSS.

**Types of Deviant behaviors**	**Mixed Schools**	${\overset{¯}{X}}_{M}$	**Girls’ Schools**	${\overset{¯}{X}}_{G}$	**Boys Schools**	${\overset{¯}{X}}_{B}$	**Total**	**Overall**$\overset{¯}{X}$
R12	261	0.870	286	0.953	240	0.800	787	0.874
R15	237	0.790	264	0.880	196	0.653	697	0.774
R5	194	0.647	183	0.610	230	0.767	607	0.674
R6	209	0.697	161	0.537	178	0.593	548	0.609
R18	147	0.490	203	0.677	188	0.627	538	0.598
R8	170	0.567	136	0.453	188	0.627	494	0.549
R7	126	0.420	169	0.563	192	0.640	487	0.541
R16	142	0.473	151	0.503	140	0.467	433	0.481
R21	152	0.507	93	0.310	160	0.533	405	0.450
R19	107	0.357	129	0.430	111	0.370	347	0.386
R1	90	0.300	59	0.197	182	0.607	331	0.368
R14	127	0.423	78	0.260	107	0.357	312	0.347
R2	85	0.283	50	0.167	165	0.550	300	0.333
R20	90	0.300	50	0.167	89	0.297	229	0.254
R3	88	0.293	68	0.227	68	0.227	224	0.249
R4	60	0.200	52	0.173	80	0.267	192	0.213
R17	63	0.210	81	0.270	48	0.160	192	0.213
R11	72	0.240	15	0.050	47	0.157	134	0.149
R10	61	0.203	7	0.023	24	0.080	92	0.102
R13	36	0.120	11	0.037	19	0.063	66	0.073
R9	48	0.160	0	0.000	0	0.000	48	0.053

**Remarks:** Likert scale of 3 was used for the coding. Rarely is assigned ‘0’, occasionally is assigned ‘1’ and very often is assigned ‘2’. The variables with higher mean values are the most prevalent deviant behavior among the 900 respondents. ${\overset{¯}{X}}_{M}$, ${\overset{¯}{X}}_{G}$, ${\overset{¯}{X}}_{B}$ and $\overset{¯}{X}$are the means for the mixed schools, girls ‘schools, boys’ schools and total mean respectively.

**Table 2:** The data summary of analysis of DSST.

**Types of Deviant behaviors**	**Mixed Schools**	${\overset{¯}{X}}_{M}$	**Girls’ Schools**	${\overset{¯}{X}}_{G}$	**Boys Schools**	${\overset{¯}{X}}_{B}$	**Total**	**Overall**$\overset{¯}{X}$
S17	65	1.444	61	1.356	79	1.756	205	1.519
S19	54	1.200	71	1.578	64	1.422	189	1.400
S20	61	1.356	75	1.667	47	1.044	183	1.356
S1	54	1.200	46	1.022	74	1.644	174	1.289
S15	52	1.156	67	1.489	50	1.111	169	1.252
S7	61	1.356	50	1.111	56	1.244	167	1.237
S2	47	1.044	43	0.956	68	1.511	158	1.170
S3	50	1.111	51	1.133	48	1.067	149	1.104
S16	43	0.956	60	1.333	28	0.622	131	0.970
S18	31	0.689	20	0.444	69	1.533	120	0.889
S6	34	0.756	18	0.400	67	1.489	119	0.881
S8	33	0.733	39	0.867	47	1.044	119	0.881
S21	28	0.622	40	0.889	46	1.022	114	0.844
S5	32	0.711	33	0.733	42	0.933	107	0.793
S12	35	0.778	11	0.244	47	1.044	93	0.689
S11	22	0.489	19	0.422	41	0.911	82	0.607
S4	20	0.444	9	0.200	25	0.556	54	0.400
S14	9	0.200	12	0.267	32	0.711	53	0.393
S13	5	0.111	8	0.178	11	0.244	24	0.178
S9	8	0.178	2	0.044	11	0.244	21	0.156
S10	5	0.111	3	0.067	9	0.200	17	0.126

Furthermore, research questions can be posed and hypotheses tests can be obtained. Also the comparison between the analysis of the scores of DSSS and DSST can be obtained and validated by the necessary statistical tools. The questionnaires can be modified to include measures of deviant behaviors not captured in this article.

### 2.4 Incidence of deviant behavior

**Fig. 1:**
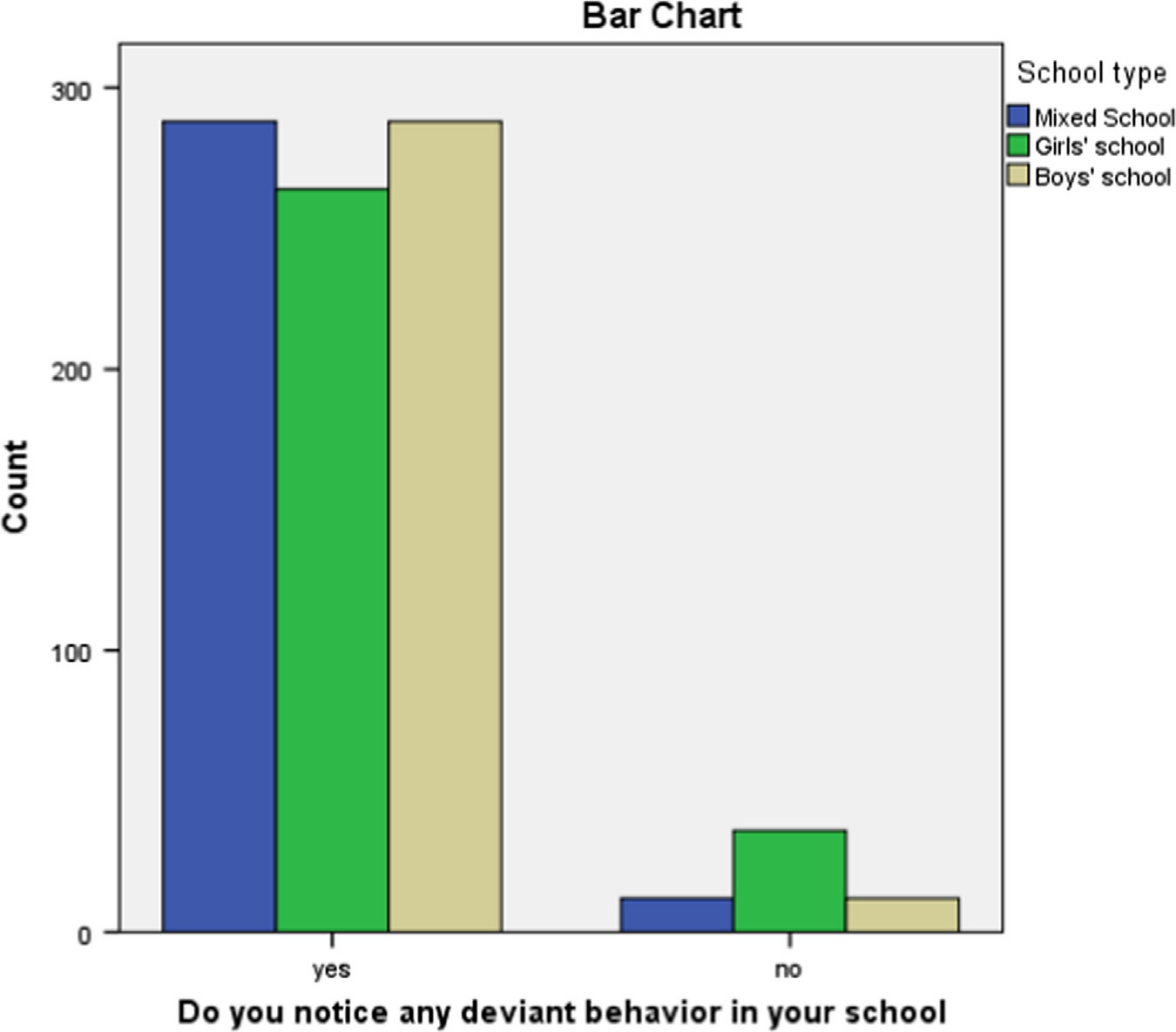
Perceived incidence of Deviant behavior as responded by the students.

**Fig. 2:**
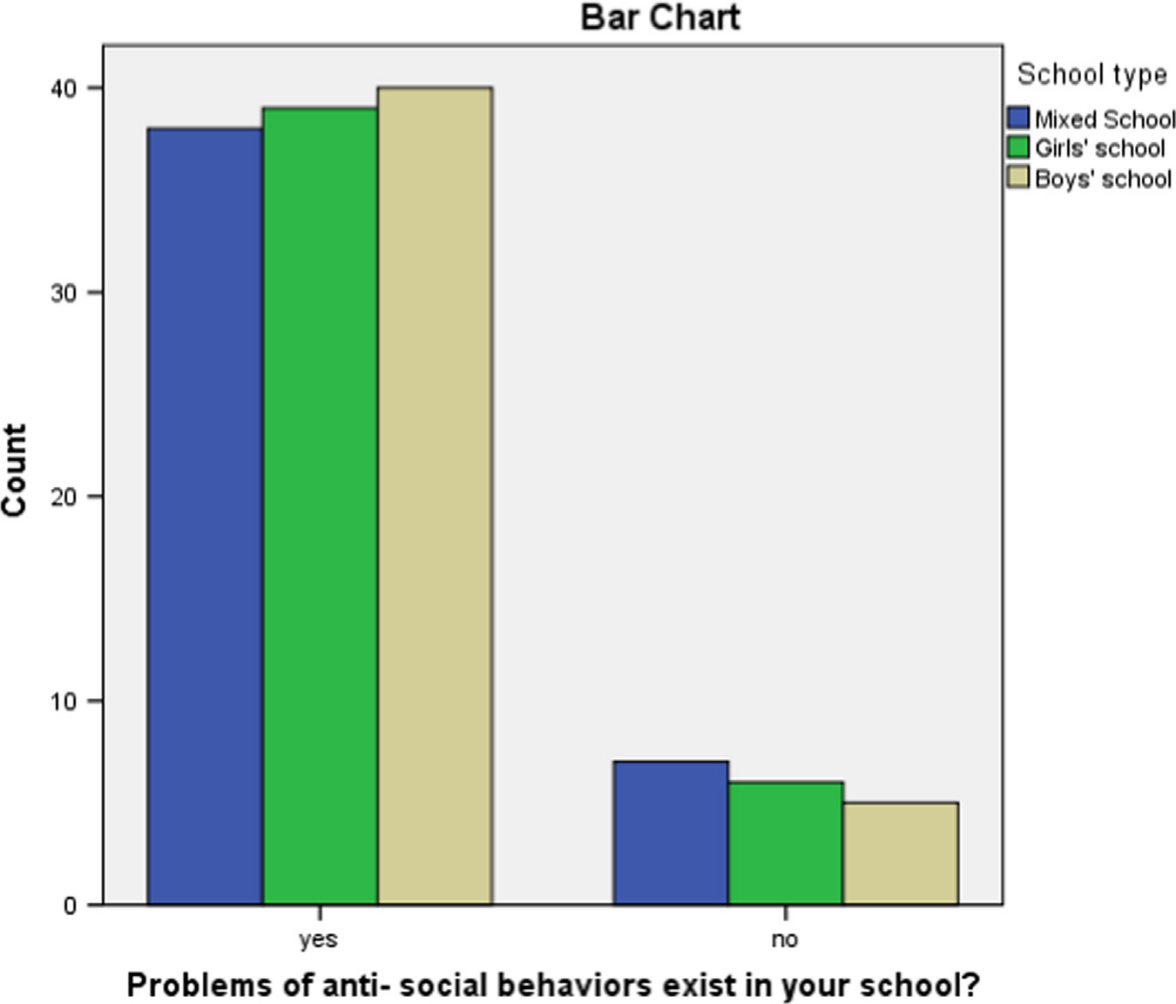
Perceived incidence of Deviant behavior as responded by the teachers.

There seems to be a general agreement of the prevalence and incidence of deviant behaviors as observed by both the students and the teachers. These are presented in Fig. 1, Fig. 2.
