# Supplementary material for: Survey dataset on the types, prevalence and causes of deviant behavior among secondary school adolescents in some selected schools in Benin City, Edo State, Nigeria
Source: Data Brief. 2018 Jul 27;20:101–7. doi: 10.1016/j.dib.2018.07.059 (PMC6088562; doi:10.1016/j.dib.2018.07.059)
Supplement: Supplementary file 1 — Supplementary material [file mmc1.docx]

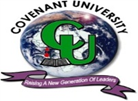


**COVENANT UNIVERSITY, OTA NIGERIA**

**COLLEGE OF SCIENCE AND TECHNOLOGY**

**MATHEMATICS DEPARTMENT**

**CONFLICT OF INTEREST**

All the authors made some valuable contributions and state that there are no competing interests in the publication of this research work

Thank you

Signed: Sheila A. Bishop

4^th^ June, 2018
