# Supplementary material for: Survey dataset on the types, prevalence and causes of deviant behavior among secondary school adolescents in some selected schools in Benin City, Edo State, Nigeria
Source: Data Brief. 2018 Jul 27;20:101–7. doi: 10.1016/j.dib.2018.07.059 (PMC6088562; doi:10.1016/j.dib.2018.07.059)
Supplement: Supplementary file 2 — Supplementary material [file mmc2.zip › Supplementary DATA/Supplementary Data A.docx]

**STUDENTS QUESTIONNAIRE**

The purpose of this questionnaire is to solicit responses that will help the researcher to determine the factors responsible for the types and prevalence of students’ behavior in our secondary schools in Benin City.

All information gathered in the questionnaire will be treated with utmost confidentiality. To guarantee this, your name is not required.

Indicate your response by ticking the appropriate box.

Sex. Male / / Female / /

Age (years). 9-11 / / 12-14 / / above 15 / /

School type: Mixed School / / Girls’ School / / Boys’ School / /

**SECTION A**

Tick only one response either ‘Yes’ or ‘No’

1. Do you notice any deviant behavior in your school? Yes / / No / /
2. If you agree, tick the forms/types of deviant behaviors you have observed and the degree of their occurrence in your school.

| **Rarely** |  | **Very Often** |
| --- | --- | --- |
| **Occasionally** |  |  |

1. Rudeness to authority
2. Disobedience to rules and

regulations

1. Lying to avoid being disciplined
2. Stealing when my own thing is stole
3. Fight often to defend myself
4. Truancy when the class is boring
5. Lateness to school
6. Intolerant of junior students
7. Can smoke if given opportunity
8. Cheating when sent on errands
9. Bullying
10. Often stand on my desk
11. Getting help from others during exams
12. Absconding from home/school
13. Screaming in class
14. Forcing people to understand me
15. Stay longer playing at break
16. Often got injured while playing
17. Hate doing assignments or practical
18. Speaking loud when angry
19. Often use force on others

**SECTION B**

Indicate by ticking either ‘Yes’ or ‘No’ the likely causes for the prevalence of the types of deviant behaviors you have ticked in **SECTION A** from the following questions.

1. The characteristics of teachers such as mode of dressing, relationship with others, attitude to work, use of foul language, etc. contributing to some deviant behaviors. Yes / / No / /
2. The characteristics of parents such as mode of dressing, excessive drinking of alcohol, smoking, lack of proper parental care, etc. that can influence the prevalence of deviant behaviors.

Yes / / No / /

1. Exclusion of student’s representatives while taking decisions on matters affecting their well-being can lead to rioting, disobedience, break of law and order, etc. Yes / / No / /
2. Exposure to pornographic materials through social media, magazines, literature, etc. can lead to prevalence of some deviant behaviors ticked in section A. Yes / / No / /
3. The increase in corruption in the society may be responsible for the prevalence of some deviant behaviors in our schools. Yes / / No / /
4. Lack of guidance and counselling may be responsible for the prevalence of some deviant behaviors in our schools. Yes / / No / /
5. Do my parents encourage me been punished for my wrongful acts? Yes / / No / /
6. Do poor financial support from your parents contributes to the prevalence of deviant behaviors? Yes / / No / /
7. How often are you punished for committing oftenest that negates school rules and regulations?
8. Often / /
9. Rarely / /
10. Occasionally / /
