# Supplementary material for: Survey dataset on the types, prevalence and causes of deviant behavior among secondary school adolescents in some selected schools in Benin City, Edo State, Nigeria
Source: Data Brief. 2018 Jul 27;20:101–7. doi: 10.1016/j.dib.2018.07.059 (PMC6088562; doi:10.1016/j.dib.2018.07.059)
Supplement: Supplementary file 2 — Supplementary material [file mmc2.zip › Supplementary DATA/Supplementary Data B.docx]

**TEACHER’S QUESTIONNAIRE**

This questionnaire seeks to find out your honest opinion about some of the things that happens constantly in your school.

All information gathered in the questionnaire will be treated with utmost confidentiality. To guarantee this, your name is not required.

Indicate your response by ticking the appropriate box like this / X /

Sex. Male / / Female / /

Age: Below 30 / / Between 30 and 40 / / Above 40 / / Years

School type: Mixed School / / Girls’ School / / Boys’ School / /

**SECTION A**

Tick only one response either ‘Yes’ or ‘No’ ‘Rarely’ or ‘Occasionally’ or ‘Very often’\

1. Problems of anti- social behaviors exist in your school? Yes / / No / /
2. If you agree, tick the forms of anti- social behaviors which you have observed with the degree of their occurrence among adolescents in your school.

| **Rarely** | **Occasionally** | **Very Often** |
| --- | --- | --- |
|  |  |  |

Have you noticed students exhibiting any of the behaviors?

1. Rudeness to authority
2. Disobedience to rules and regulations
3. Lying to avoid being disciplined
4. Stealing when their things are stolen
5. Fight often to defend themselves
6. Truancy when the class is boring
7. Lateness to school
8. Aggression towards junior students
9. Notice that the student has the tendency to smoke
10. Cheating when sent on errands
11. Bullying
12. Standing on their desks
13. Getting help from others during exams
14. Absconding from home/school
15. Screaming in class
16. Forcing people to understand them
17. Stay longer playing at break
18. Often got injured while playing
19. Hate doing assignments or practical
20. Speaking loud when angry
21. Using force on others

**SECTION B**

Indicate by ticking either ‘Yes’ or ‘No’ the likely causes for the prevalence of the types of deviant behaviors you have ticked in **SECTION A** from the following questions.

1. The characteristics of teachers such as mode of dressing, relationship with others, attitude to work, use of foul language, etc. contributing to some deviant behaviors. Yes / / No / /
2. The characteristics of parents such as mode of dressing, excessive drinking of alcohol, smoking, lack of proper parental care, etc. that can influence the prevalence of deviant behaviors.

Yes / / No / /

1. Exclusion of student’s representatives while taking decisions on matters affecting their well-being can lead to rioting, disobedience, break of law and order, etc. Yes / / No / /
2. Exposure to pornographic materials through social media, magazines, literature, etc. can lead to prevalence of some deviant behaviors ticked in section A. Yes / / No / /
3. The increase in corruption in the society may be responsible for the prevalence of some deviant behaviors in our schools. Yes / / No / /
4. Lack of guidance and counselling may be responsible for the prevalence of some deviant behaviors in our schools. Yes / / No / /
5. Do some parents discourage the school authority from punishing their children who have committed offense? Yes / / No / /
6. Poor financial support from parents contributes to the prevalence of deviant behaviors? Yes / / No / /
7. What means of punishments are used to stop, correct and discourage the prevalence of some of these deviant behaviors?
8. Corporal punishment / /
9. Suspension / /
10. Expulsion from school / /
11. Guidance and counseling / /
12. What are your recommendations for solving problems of deviant behaviors mentioned above, so as to stop further occurrence in our schools and society at large?
13. …………………………………………………………………
14. ………………………………………………………………….
15. …………………………………………………………………
16. …………………………………………………………………
